# Electronic Cigarette Use and the Diagnosis of Nonmelanoma Skin Cancer Among United States Adults

**DOI:** 10.7759/cureus.19053

**Published:** 2021-10-26

**Authors:** Katelyn Dugan, Shelby Breit, Hayrettin Okut, Elizabeth Ablah

**Affiliations:** 1 Department of Population Health, University of Kansas School of Medicine-Wichita, Wichita, USA

**Keywords:** tobacco adverse effects, e-cigarette smoking, electronic cigarette, nonmelanoma skin cancer risk, nonmelanoma

## Abstract

Introduction

Electronic cigarette (e-cigarette) use has many potential effects, including damage to the skin. Limited research has assessed e-cigarette use with the incidence of nonmelanoma skin cancer. The current study was conducted to determine if a relationship exists between nonmelanoma skin cancer diagnosis and e-cigarette use among US adults.

Methods

Data from the National Health Interview Survey (NHIS) were used to assess if a relationship exists between e-cigarette use and the diagnosis of nonmelanoma skin cancer. Data within the sample adult files for years 2014 through 2018, along with the adult cancer file for the year 2015, were analyzed. Participants who reported having a diagnosis of nonmelanoma skin cancer were included. Participants who reported never being diagnosed with any type of cancer were included as a comparison group.

Results

Males and those of older age were significantly more likely to be diagnosed with nonmelanoma skin cancer compared to females and younger adults. Cigarette use was significantly associated with an increased risk of nonmelanoma skin cancer. There was no relationship between e-cigarette use and diagnosis of nonmelanoma skin cancer.

Conclusions

Although the current study did not find an association between e-cigarette use and nonmelanoma skin cancer diagnosis, a longer study period and larger sample size may more clearly determine if an association exists.

## Introduction

In the United States, the incidence of nonmelanoma skin cancer, which includes both squamous cell carcinoma (SCC) of the skin and basal cell carcinoma (BCC), increased from more than 4 million in 2006 to more than 5.4 million in 2012 [1]. However, the incidence of nonmelanoma skin cancer may be greater, as it is not mandatory for physicians to report nonmelanoma cases to a national cancer registry [2]. While nonmelanoma skin cancer incidence may be increasing, cigarette smoking among US adults has declined in recent years [3] and electronic cigarette (e-cigarette) use is increasing. In 2018, 3.2% of US adults (8.1 million) were suggested to be current users of e-cigarettes, which was an increase from 2.8% in 2017 [4]. E-cigarettes may have fewer toxic substances than traditional cigarettes and have therefore been claimed to be a healthier alternative with perceived health benefits by electronic cigarette users [5-7]. The use of e-cigarettes has also been suggested to be helpful for traditional cigarette smoking cessation [8-10]. However, in regards to the skin, vapor from e-cigarettes has been shown to be toxic to human skin cells, causing cell death consistent with that caused by cigarette smoke [11]. Still, the harms of e-cigarette use are not fully known, including potential damage to the skin. Limited research has assessed e-cigarette use with the incidence of nonmelanoma skin cancer. The current study was conducted to determine if a relationship exists between nonmelanoma skin cancer diagnosis and e-cigarette use among US adults.

## Materials and methods

Participants

Data for this study were collected from the National Health Interview Survey (NHIS) [12]. The NHIS is a household survey conducted throughout each year to collect information about US noninstitutionalized populations. Certain groups are excluded from the survey, including people living in long-term care facilities. College dormitories were sampled in the years 2016 and 2017. As part of the NHIS, one adult per family is selected to participate in the Sample Adult Core questionnaire. Black, Hispanic, or Asian adults aged 65 years or older had a greater chance than other family members to be asked to participate in the Sample Adult Core questionnaire, with this sample design used until 2019. For this study, data within the sample adult files for years 2014 through 2018, along with the adult cancer file for the year 2015, were analyzed. Participants who reported having a diagnosis of nonmelanoma skin cancer were included. To establish a comparison group, participants who reported never being diagnosed with any type of cancer were also included.

Instrument

The Sample Adult questionnaire included demographic information, such as region, race, sex, and ethnicity, as well as information about prior cancer diagnoses and exposures such as cigarette and e-cigarette use. In 2015, information regarding e-cigarette use was in the Sample Adult Cancer file. Information from this file was merged with information from the 2015 sample adult file. Up to three types of cancer diagnoses could be documented per participant. If a participant had received more than three types of cancer diagnoses, it was documented, but further types were not specified. To determine the use of e-cigarettes, responses to “ever used electronic cigarettes” were used, and to determine the use of combustible cigarettes, responses to “smoked 100 cigarettes” were used. Cigarette use, sex, and age were controlled for in the multivariate model.

Analysis

The current study was determined to be “not human subjects research” by the University of Kansas Medical Center’s Human Research Protection Program. Statistical analysis was conducted using SAS 9.4 (SAS Inc, Carry, NC). Categorical variables were presented as frequencies and percentages, and continuous variables were presented as means ± standard deviation (SD). The associations between categorical variables were tested by Pearson's chi-square and likelihood chi-square. PROC SURVEYLOGISTIC in SAS (SAS/STAT, version 15.1) was used to test the association between nonmelanoma skin cancer diagnosis and e-cigarette use after controlling the confounding variables. Taylor series linearization approach was considered to estimate the variance of weighted regression coefficients. A two-sided p-value ≤ 0.05 was considered statistically significant.

## Results

Of the 155,556 total respondents, 2.2% (n = 3,454) reported being diagnosed with nonmelanoma skin cancer. These participants were interviewed throughout the US, with 34.8% (n = 1,202) living in the South, 26.3% of participants (n = 909) in the West, 22.8% of participants (n = 786) in the Midwest, and 16.1% of participants (n = 557) in the Northeast (Table 1). Many more respondents, 89.4% (n = 138,991), reported never being diagnosed with any type of cancer. Participants never diagnosed with cancer were also interviewed throughout the US, with 35.6% (n = 49,437) living in the South, 26.0% (n = 36,145) living in the West, 22.1% (n = 30,673) living in the Midwest, and 16.4% (n = 22,736) living in the Northeast (Table 2). Most participants who reported having had nonmelanoma skin cancer were White (98.5%, n = 3,403), non-Hispanic (98.2%, n = 3,391), and female (52.2%, n = 1,803) (Table 1). Most participants never diagnosed with any type of cancer were also White (73.7%, n = 102,407), non-Hispanic (84.9%, n = 118,023), and female (54.5%, n = 75,730) (Table 2). The mean age of the participants who had non-melanoma skin cancer was 67 years, whereas the mean age of participants never diagnosed with any type of cancer was 49 years (Table 3).

**Table 1:** Demographics of participants diagnosed with nonmelanoma skin cancer.

	Frequency	Percent
Participants diagnosed	3,454	2.2%
Participants by region		
South	1,202	34.8%
West	909	26.3%
Midwest	786	22.8%
Northeast	557	16.1%
Race		
White	3,403	98.5%
Ethnicity		
Non-Hispanic	3,391	98.2%
Sex		
Female	1,803	52.2%

**Table 2:** Demographics of participants never diagnosed with cancer.

	Frequency	Percent
Participants never diagnosed	138,991	89.4%
Participants by region		
South	49,437	35.6%
West	36,145	26.0%
Midwest	30,673	22.1%
Northeast	22,736	16.4%
Race		
White	102,407	73.7%
Ethnicity		
Non-Hispanic	118,023	84.9%
Sex		
Female	75,730	54.5%

**Table 3:** Participants' mean age.

Diagnosed with nonmelanoma skin cancer	67 years
Never diagnosed with cancer	49 years

Among those diagnosed with nonmelanoma skin cancer, almost half (47.3%, n = 1,630) reported having smoked at least 100 cigarettes, and 7.2% (n = 247) reported having used an electronic cigarette at least one time. Of those never diagnosed with any type of cancer, 38.8% (n = 53,687) reported having smoked at least 100 cigarettes, and 14.4% (n = 19,646) reported having used an electronic cigarette at least one time (Table 4). Males and those of older age were also significantly more likely to be diagnosed with nonmelanoma skin cancer compared to females and those younger adults. Cigarette use was also significantly associated with an increased risk of nonmelanoma skin cancer. There was no relationship between e-cigarette use and diagnosis of nonmelanoma skin cancer (Table 5).

**Table 4:** Participants' cigarette and e-cigarette usage.

	Frequency	Percent
Participants diagnosed with nonmelanoma skin cancer		
Smoked at least 100 cigarettes	1,630	47.3%
Used an electronic cigarette at least one time	247	7.2%
Participants never diagnosed with cancer		
Smoked at least 100 cigarettes	53,687	38.8%
Used an electronic cigarette at least one time	19,646	14.4%

**Table 5:** Risk of nonmelanoma skin cancer.

	Odds ratio estimate	95% confidence interval	
Sex (male vs. female)	1.267	1.179	1.362
Age	1.067	1.065	1.069
Cigarette use (yes vs. no)	1.105	1.026	1.189
E-cigarette use (yes vs. no)	0.958	0.832	1.103

## Discussion

In the current study, almost all patients diagnosed with nonmelanoma skin cancer were White and non-Hispanic. This finding is consistent with another study, which suggested that 96.3% of nonmelanoma skin cancer cases were among Caucasians, and 2.9% of cases were among Hispanic Americans [13]. The current study suggests that males generally have an increased risk of developing nonmelanoma skin cancer. Another study suggests that the risk of nonmelanoma skin cancer may vary by gender, as male smokers were suggested to have a lesser risk of BCC with no change in risk of SCC, and female smokers have a greater risk of both BCC and SCC compared to those who had never smoked [14]. However, the current study did not analyze the risk for each gender regarding smoking status or type of nonmelanoma skin cancer diagnosis.

The current study suggests combustible cigarette use may be a risk factor for developing nonmelanoma skin cancer. These results are partially consistent with the results of a meta-analysis which suggested smoking can increase the risk for developing squamous cell carcinoma (SCC) of the skin and does not impact basal cell carcinoma (BCC) risk [15]. The current study suggests only a small proportion (7.2%) of those diagnosed with nonmelanoma skin cancer have ever used an e-cigarette. This is less than 12% of US adults estimated to have used an e-cigarette at least once [16]. E-cigarette use among adults has been suggested to be most prevalent among those who are younger, male, employed, in poverty, and without any college education [17].

Although our study identified no association between nonmelanoma skin cancer and only e-cigarette use, the use of e-cigarettes may still have detrimental effects on the skin. One review suggested that e-cigarettes are associated with several conditions involving the skin, including contact dermatitis, thermal injury, hyperplastic candidiasis, nicotine stomatitis, and other oral mucosal lesions [18]. There were several limitations of this study. The survey questions used captured data on any e-cigarette use but did not further specify the precise amount of e-cigarette use. Besides the amount used, the newness of e-cigarettes and the length of time that may be required for the health effects of e-cigarettes on the skin to manifest might have limited the association between e-cigarette use and nonmelanoma skin cancer diagnosis. Different results may be found over time after individuals experience longer exposure periods to e-cigarette vapor. Also, because the study design was based upon survey results, the current study could be limited by recall bias, as participants self-reported their diagnoses and behaviors. However, strengths of using the NHIS data included the large sample size provided from the US population.

## Conclusions

In the current study, cigarette use was associated with increased odds of a diagnosis of nonmelanoma skin cancer. E-cigarette use was not significantly associated with the diagnosis of nonmelanoma skin cancer. Although the current study did not find an association between e-cigarette use and nonmelanoma skin cancer diagnosis, a longer study period and larger sample size may more clearly determine if an association exists. In the future, more research will be needed to better understand the long-term health effects of e-cigarette use, including effects on the skin.

## References

[1] Rogers HW, Weinstock MA, Feldman SR, Coldiron BM (2015). Incidence estimate of nonmelanoma skin cancer (keratinocyte carcinomas) in the U.S. population, 2012. JAMA Dermatol.

[2] Gordon R (2013). Skin cancer: an overview of epidemiology and risk factors. Semin Oncol Nurs.

[3] Wang TW, Asman K, Gentzke AS (2018). Tobacco product use among adults - United States, 2017. MMWR Morb Mortal Wkly Rep.

[4] Creamer MR, Wang TW, Babb S (2019). Tobacco product use and cessation indicators among adults - United States, 2018. MMWR Morb Mortal Wkly Rep.

[5] Caponnetto P, Campagna D, Papale G, Russo C, Polosa R (2012). The emerging phenomenon of electronic cigarettes. Expert Rev Respir Med.

[6] Etter JF, Bullen C (2011). Electronic cigarette: users profile, utilization, satisfaction and perceived efficacy. Addiction.

[7] Patel D, Davis KC, Cox S (2016). Reasons for current e-cigarette use among U.S. adults. Prev Med.

[8] Hartmann-Boyce J, McRobbie H, Butler AR (2021). Electronic cigarettes for smoking cessation. Cochrane Database Syst Rev.

[9] Selya AS, Dierker L, Rose JS, Hedeker D, Mermelstein RJ (2018). The role of nicotine dependence in e-cigarettes' potential for smoking reduction. Nicotine Tob Res.

[10] Hajek P, Phillips-Waller A, Przulj D (2019). E-cigarettes compared with nicotine replacement therapy within the UK Stop Smoking Services: the TEC RCT. Health Technol Assess.

[11] Cervellati F, Muresan XM, Sticozzi C (2014). Comparative effects between electronic and cigarette smoke in human keratinocytes and epithelial lung cells. Toxicol In Vitro.

[12] (2020). National Center for Health Statistics. National Health Interview Survey, 2014 - 2018. Public-use data file and documentation. https://www.cdc.gov/nchs/nhis/data-questionnaires-documentation.htm.

[13] Loh TY, Ortiz A, Goldenberg A, Brian Jiang SI (2016). Prevalence and clinical characteristics of nonmelanoma skin cancers among Hispanic and Asian patients compared with White patients in the United States: a 5-year, single-institution retrospective review. Dermatol Surg.

[14] Song F, Qureshi AA, Gao X, Li T, Han J (2012). Smoking and risk of skin cancer: a prospective analysis and a meta-analysis. Int J Epidemiol.

[15] Leonardi-Bee J, Ellison T, Bath-Hextall F (2012). Smoking and the risk of nonmelanoma skin cancer: systematic review and meta-analysis. Arch Dermatol.

[16] Schoenborn CA, Gindi RM (2015). Electronic cigarette use among adults: United States, 2014. NCHS Data Brief.

[17] Stallings-Smith S, Ballantyne T (2019). Ever use of e-cigarettes among adults in the United States: a cross-sectional study of sociodemographic factors. Inquiry.

[18] Visconti MJ, Ashack KA (2019). Dermatologic manifestations associated with electronic cigarette use. J Am Acad Dermatol.

